# Efficacy of *Rytigynia senegalensis* Blume on Free Radical Scavenging, Inhibition of *α*-Amylase and *α*-Glucosidase Activity, and Blood Glucose Level

**DOI:** 10.1155/2022/9519743

**Published:** 2022-09-27

**Authors:** Barthelemy Maidadi, Fidèle Ntchapda, David Miaffo, Oulianovie Kamgue Guessom

**Affiliations:** ^1^Department of Biological Sciences, Faculty of Sciences, University of Maroua, P.O. Box 814, Maroua, Cameroon; ^2^Department of Biological Sciences, Faculty of Science, University of Ngaoundere, P.O. Box 454, Ngaoundere, Cameroon; ^3^Department of Life and Earth Sciences, Higher Teachers' Training College, University of Maroua, P.O. Box 55, Maroua, Cameroon; ^4^Department of Animal Science, Faculty of Agriculture and Veterinary Medicine, University of Buea, P.O. Box 63, Buea, Cameroon

## Abstract

*Rytigynia senegalensis* (Rubiaceae) is a plant used in African medicine for the treatment of diabetes. The aim of this study was to evaluate the *in vitro* antioxidant, enzyme inhibitory, and hypoglycemic effects of *Rytigynia senegalensis* extract (RSE). The contents of phenols, tannins, and flavonoids were determined by phytochemical screening. 2,2-Azino-bis-3-ethylbenzothiazoline-6-sulfonic acid (ABTS) and 2,2-diphenylpicrylhydrazyl (DPPH) were determined to evaluate the free radical scavenging capacity of the RSE. The inhibitory activity of *α*-amylase and *α*-glucosidase was evaluated *in vitro* using the *α*-amylase and *α*-glucosidase inhibition methods and *in vivo* using the sucrose and starch tolerance tests. The glucose tolerance test was performed on normal rats using doses of 50, 100, and 200 mg/kg of RSE. RSE contains total phenols (36.35 mg GAE/g of extract), flavonoids (11.91 mg QE/g of extract), and tannins (13.01 mg CE/g of extract). RSE exhibits significant radical scavenging activity on DPPH and ABTS radicals with an IC_50_ of 17.51 and 21.89 *μ*g/mL, respectively. RSE showed an inhibitory effect on the activity of *α*-amylase and *α*-glucosidase with an IC_50_ of 308.93 and 354.13 *μ*g/mL, respectively. RSE (100 and 200 mg/kg) caused a significant decrease in area under the curve and postprandial glycemia at 60, 90, and 120 min following the administration of starch or sucrose. Regarding the glucose tolerance test, RSE (100 and 200 mg/kg) significantly reduced postprandial hyperglycemia from the 90th min posttreatment. RSE lowered postprandial hyperglycemia and has antioxidant properties. These effects would be due to the presence of bioactive compounds in the RSE.

## 1. Introduction

Diabetes mellitus is a metabolic disease responsible for major public health problems [1]. This disease is characterized by chronic hyperglycemia linked to a deficiency in either the secretion or the action of insulin, or both [2]. Globally, 463 million people have diabetes [3]. Predictions estimate that this prevalence will be around 550 million in 2025 and 642 million in 2040 [3]. Regarding type 2 diabetes, targeting enzymes involved in processing dietary carbohydrates in the intestinal tract is among the vital targets. Among these, *α*-amylase and *α*-glucosidase enzymes are of high pharmacological interest and are used to control elevated glucose level in type 2 diabetes mellitus [4]. For instance, *α*-amylase and *α*-glucosidase enzymes are responsible for the breakdown of starch and oligosaccharides to glucose and their inhibition plays a significant role to decrease the absorption of glucose in the intestine [5]. Long-chain carbohydrates are broken down into glucose by alpha-amylase enzyme, whereas *α*-glucosidase is responsible for the breakdown of disaccharides and starch into simpler monosaccharide glucose, resulting in hyperglycemia [6]. Untreated or poorly treated diabetes can lead to complications in blood vessels and thereby lead to cardiovascular disease, blindness, and kidney failure [7].

Some pharmaceutical treatments decrease the impact of the disease and its complications. The aim of these treatments is to reduce hyperglycemia by activating the endogenous secretion of insulin with sulphonylureas and glitinides or by improving the sensitivity of target tissues to insulin with thiazolidinediones and biguanides [8]. Other drugs such as miglitol and acarbose have inhibitory effects on the activity of *α*-amylase and *α*-glucosidase enzymes, mainly indicated in postprandial hyperglycemia. They can be used as monotherapy or in addition to insulin and other oral antidiabetic drugs [9]. Regardless of how they are administered, these medicines have harmful side effects such as gas, digestive pain, and diarrhea. In addition, they are contraindicated in patients at risk whose digestive system is too fragile to adapt to the disturbance caused by their mechanism of action. Given all of these limitations, more than 80% of the population resort to traditional medicine to treat common illnesses [10]. The use of medicinal plants and natural products is still a major source of therapy in the developing countries [11, 12]. The discovery of modern analytical techniques has further eased the process of ethnomedicinal drug discovery to identify, isolate, and characterize target molecules [13, 14]. Approximately more than four hundred plants are identified as having antidiabetic potential, but only a few of these plants have received medical and scientific evaluation [15]. A large number of *α*-amylase and *α*-glucosidase inhibitors are produced by different microorganisms and plants to regulate the activities of these enzymes [16]. The natural *α*-glucosidase inhibitors from plant sources, whose *α*-glucosidase inhibitory activities have been reported previously, include alkaloids, flavonoids, anthocyanins, terpenoids, curcuminoids, and phenolic compounds [17]. For instance, anthocyanins isolated from *Pharbitis nil* L. and *Ipomoea batatas* L. significantly reduce postprandial hyperglycemia via inhibition of*α* glucosidase enzyme [18].

Hyperglycemia is often accompanied by the accumulation of free radicals, which contributes to severe complications in diabetics [19]. Herbs with both antihyperglycemic and anti-free radical properties may be beneficial in the management of diabetes mellitus and its complications. R. *senegalensis* Blume is a glabrous shrub with slender branchlets, more or less scrambling, with elegant foliage; flowers are white or greenish, paired on slender axillary peduncles, with reflexed petals. It is a plant of the Rubiaceae family, found in Africa in the Sahelian areas, and used empirically to treat hemorrhoids, constipation, pain during childbirth, hypertension, dysentery, and diabetes [20]. To the best of our knowledge, no previous study has been done on its antidiabetic and anti-free radical properties. The aim of this study was therefore to evaluate the anti-free radical and antihyperglycemic potential of RSE.

## 2. Materials and Methods

### 2.1. Chemicals

Acarbose, *α*-glucosidase, *α*-amylase, 3,5-dinitrosalicyclic acid, and p-nitrophenyl-*α*-D-glucopyranoside were purchased from Sigma-Aldrich, Saint, USA. Amidon, D-glucose, and sucrose were purchased from EduLab Biology Kit, Bexwell, Norfolk, PE38 9 GA, UK. These drugs and chemicals were obtained commercially in analytical grade.

### 2.2. Animal Material

The animal used consisted of male rats of the Wistar strain, aged between 10 and 12 weeks and weighing between 220 and 250 g. These rats were provided by the Laboratory of Medicinal Plants, Health and Galenic Formulation of the Faculty of Sciences of the University of Ngaoundéré (Cameroon) in polystyrene cages under conditions of ambient temperature (25 ± 2°C) and natural light (12 h light-dark cycle). Food and drinking water were given ad libitum for the duration of the experiment. The animals were acclimatized under laboratory conditions for 14 days before the various tests. Experimental animals were treated according to the Cameroon National Ethics Committee (Ref. no. FWIRB 00001954), and all experiments were reviewed and approved.

### 2.3. Plant Material

*R*. *senegalensis* was collected in the locality of Pette, a village located about 70 km of Maroua, Cameroon. A sample of it was identified by the botanist Dr. Todou Gilbert and kept at the National Herbarium of Cameroon under number 4496/SRFK. The harvested plant was washed with tap water, dried in the shade at room temperature, and then reduced to a fine powder.

### 2.4. Preparation of the Extract

A mass of 14.28 g of powder of *R*. *senegalensis* was introduced into 500 mL of distilled water previously brought to the boil for 30 min. After cooling, the solution was filtered through Whatman No. 1 paper. The filtrate obtained was evaporated in an oven at 45°C for 48 h, which allowed us to obtain 1.01 g of crude extract, i.e., a yield of 7.07%.

### 2.5. Qualitative Phytochemical Screening

Qualitative phytochemical tests were carried out according to the method described by Gilbert and Pratley [21].

#### 2.5.1. Test for Tannins

The appearance of bluish-green coloration on the addition of 2 mL of RSE (1 mg/mL) to 3–4 drops of ferric chloride solution (5%) indicated the presence of tannins.

#### 2.5.2. Tests for Phenols

RSE (2 mL) was prepared and then 5% of ferric chloride solution is added. The presence of phenols in the RSE is marked by the appearance of the dark green color.

#### 2.5.3. Tests for Flavonoids

The existence of the flavonoids was confirmed by the appearance of pink color after a few minutes when small pieces of magnesium ribbon and concentrated HCl were combined with RSE.

#### 2.5.4. Test for Saponins

RSE (1 mL), distilled water (5.0 mL), and a few drops of oil were mixed in a test tube and vigorously shaken. The appearance of a foam of about 1 cm indicated the presence of saponins in the RSE.

#### 2.5.5. Test for Quinones

A small amount of RSE was treated with concentrated HCL and observed for the formation of yellow precipitation (or coloration).

#### 2.5.6. Test for Terpenoids

The terpenoids were indicated via gray color formation when 2 mL of chloroform was added with the 5 mL of RSE, and then, 3 mL of H_2_SO_4_ concentrated was added and boiled.

#### 2.5.7. Test for Steroids

5 mL of RSE was added with 2 mL of H_2_SO_4_ concentrated and chloroform. The appearance of red color marks the presence of steroids in the RSE.

#### 2.5.8. Test for Alkaloids

RSE was dissolved in dilute hydrochloric acid and filtered. The presence of alkaloids was confirmed by a yellow-colored precipitate when filtrates of RSE were treated with potassium mercuric iodide (Mayer's reagent).

#### 2.5.9. Tests for Glycosides

RSE was added with 2 ml of acetic acid and 2 mL of chloroform. The mixture was then cooled, and H_2_SO_4_ concentrated was added. The appearance of the green color is proof of the presence of glycosides in the RSE.

### 2.6. Quantitative Phytochemical Screening

#### 2.6.1. Total Phenol Content

A mixture of 0.5 mL of a gallic acid or RSE solution (0 to 100 *μ*g/mL), 2.5 mL of the Folin-Ciocalteu reagent (10%), and 4 mL of sodium carbonate solution (7.5%, *w/v*) were incubated for 30 min at room temperature [22]. The absorbance of the mixture was read at a wavelength of 727 nm. The total phenol content of the sample was expressed in milligrams of gallic acid equivalents per gram (mg GAE/g) of dry extract.

#### 2.6.2. Total Flavonoid Content

A mixture of 1 mL of quercetin or RSE solution (0 to 100 *μ*g/mL), 0.2 mL of aluminum chloride solution (10% *w/v*), 0.2 mL of potassium acetate solution (1 M), and 5.6 mL of distilled water were incubated for 30 min at room temperature [23]. The absorbance of the mixture was read at a wavelength of 415 nm. The total flavonoid content of the sample was expressed in milligrams of quercetin equivalents per gram (mg QE/g) of dry extract.

#### 2.6.3. Total Tannin Content

A mixture of 50 *μ*L of catechin or RSE solution (0 to 100 *μ*g/mL), 750 *μ*L of chloride acid solution (12 M), and 1.5 mL of methanol solution (4%) were incubated for 20 min at room temperature [24]. The absorbance of the mixture was read at a wavelength of 500 nm. The total tannin content of the sample was expressed in milligrams of catechin equivalent per gram (mg CE/g) of dry extract.

### 2.7. Free Radical Scavenging Potential of RSE

#### 2.7.1. Diphenyl-1-Picrylhydrazyl (DPPH) Radical Scavenging Activity

RSE solution (2.5 mL) at different concentrations (1–200 *μ*g/mL) was mixed with 0.5 mL of freshly DPPH solution (0.2 mM in 1 mL of ethanol), and the mixture was vigorously vortexed at room temperature for 30 min [25]. RSE was replaced with distilled water in the control tubes, and the positive control used was Trolox. The absorbance of the mixture was read at a wavelength of 517 nm. The antiradical activity of the RSE was expressed as a percentage inhibition (I%) according to the following equation: I% = ((absorbance control − absorbance sample)/absorbance control) × 100. The amount of the sample necessary to decrease the absorbance of DPPH by 50% (IC_50_) was calculated graphically.

#### 2.7.2. 2,2-Azino-Bis-3-Ethylbenzothiazoline-6-Sulphonic Acid (ABTS) Radical Scavenging Assay

The method described by Re et al. [26] allowed us to determine the ABTS radical scavenging activity of RSE. The ABTS solution was prepared by mixing 5 mL of ABTS (7 mM) and 5 mL of potassium persulfate solution (2.4 mM) and allowed to react in the dark at room temperature for 12 h. Before use, ABTS^+^ solution (1 mL) was diluted with ethanol (60 mL) to an absorbance of 0.70 ± 0.05 at 734 nm. Afterward, 100 *μ*L of RSE or butylhydroxyanisole at different concentrations (1–200 *μ*g/mL) to 4 mL of diluted ABTS+ solution, absorbance was measured at 734 nm after 7 min. ABTS scavenging ability was expressed as IC_50_ (l g/mL), and the inhibition percentage was calculated using the following formula: ABTS^+^ radical scavenging activity (I%) = ((absorbance control − absorbance sample)/absorbance control) × 100.

### 2.8. Digestive Enzyme Inhibitory Potential of RSE

#### 2.8.1. *In Vitro* Inhibition Test of *α*-Amylase Activity

Briefly, 100 *μ*L of RSE or acarbose solution (25, 50, 100, 200, and 300 *μ*g/mL) and 100 *μ*L of *α*-amylase solution (0.5 mg/mL in 0.20 mM sodium phosphate buffer, pH 6.9) were mixed and preincubated for 10 min at 25°C [4]. Afterward, 200 *μ*L of 1% starch solution in 0.02 M sodium phosphate buffer (pH 6.9) was added to the mixture. After incubation at 37°C for 10 min, the reaction was stopped by the addition of 200 *μ*L of 3,5-dinitrosalicylic acid reagent. The tubes were cooled at room temperature after incubating them in boiling water for 5 min. Then, 5 mL of distilled water was added to the reaction mixture and absorbance was measured at 540 nm. The inhibition activity was calculated as follows: % inhibition = ((Abs control − Abs sample)/Abs control) × 100, where Abs is absorbance. Control represents 100% enzyme activity and was conducted in a similar way by replacing RSE with distilled water. For the blank, the enzyme solution was replaced with distilled water. The inhibitory activity was expressed as the concentration of the RSE or acarbose resulting in 50% inhibition of the enzyme (IC_50_) and calculated by regression analysis.

#### 2.8.2. *In Vitro* Inhibition Test of *α*-Glucosidase Activity

The inhibitory effect of the RSE on the activity of the *α*-glucosidase enzyme was determined by the method described by Kim et al. [27]. Indeed, 50 *μ*L of RSE or acarbose at different concentrations (25, 50, 100, 200, and 300 *μ*g/mL) was introduced into 100 *μ*L of Tris buffer (20 mM; pH 6.8) containing 100 *μ*L of *α*-glucosidase solution (0.01 mg/mL) and preincubated at 25°C for 10 min. After preincubation, 50 *μ*L of p-nitrophenyl-*α*-D-glucopyranoside (5 mM) was added to each tube to start the reaction and the mixture was again incubated at 37°C for 15 min. The reaction was stopped after adding 2 mL of Na_2_CO_3_ (500 mM) to each tube. The absorbance of the sample was read at 400 nm. The percent inhibition (I%) and the concentration of the RSE required to inhibit the activity of the enzyme by 50% (IC_50_) were determined as in the test for inhibition of the activity of *α*-amylase enzyme.

#### 2.8.3. *In Vivo* Inhibition Test of *α*-Amylase Activity (Starch Tolerance Test)

A starch tolerance test was performed in normal animals according to the method described by Miaffo et al. [28]. Here, 30 male rats fasted non-water for 16 hours were divided into 5 groups of 6 rats each and treated as follows: group 1 (control group) received distilled water (10 mL/kg); group 2 (reference group) treated with acarbose (10 mg/kg); groups 3, 4, and 5 received the RSE at doses of 50, 100, and 200 mg/kg, respectively. Ten minutes (10 min) after taking the initial blood sugar (*t*_0_) followed by their treatment, each rat received orally 3 g/kg of the starch solution. On the 30th, 60th, 90th, and 120th minutes following the carbohydrate overload, the blood glucose level was measured with a glucometer and strips.

#### 2.8.4. *In Vivo* Inhibition Test of *α*-Glucosidase Activity (Sucrose Tolerance Test)

The sucrose tolerance test was performed similarly to the starch tolerance test described above, with the only difference being that the sucrose solution was administered at a dose of 4 g/kg body weight [28].

### 2.9. Hypoglycemic Plus Antihyperglycemic Effects of RSE in Normal Rats

The hypoglycemic and antihyperglycemic effects of RSE were evaluated according to the method described by Kato and Miura [29] with slight modifications. Twenty-five (25) male rats were fasted for 14 hours and then divided into 5 groups of 5 rats each. The initial glycemia of the animals was evaluated in each group using a OneTouch UltraMini glucometer (Life Scan Europe, 6300 zug, Switzerland) and strips. Subsequently, the animals were treated with 10 mL/kg of distilled water (group 1), 3 mg/kg of glibenclamide (group 2), and doses of 50, 100, and 200 mg/kg of RSE (groups 3, 4, and 5, respectively). Blood glucose levels were taken at the 30th and 60th minutes posttreatment to assess the hypoglycemic effect of RSE.

Ninety minutes (90 min) after the various treatments mentioned above, the blood sugar levels of the animals were taken again. Immediately, the rats were force-fed with a solution of D-glucose (3 g/kg) and the glycemia was evaluated at times 120, 150, 180, and 210 minutes to evaluate the antihyperglycemic effect of RSE.

### 2.10. Statistical Analysis

All results were expressed as the mean ± standard derivation. The data obtained were analyzed using GraphPad Prism version 5.0 software. Two-way analysis of variance and Bonferroni's posttest were used for the treatment of double-variable tests. In contrast, one-way analysis of variance and Tukey's posttest were used to analyze data from the single-variable tests. Statistical significance was considered at *p* < 0.05.

## 3. Results

### 3.1. Qualitative Phytochemical Screening

The qualitative phytochemical study reveals the presence in RSE of bioactive compounds such as tannins, phenols, flavonoids, saponins, quinones, terpenoids, and glycosides and the absence of alkaloids, and steroids (Table 1).

### 3.2. Quantitative Phytochemical Screening

The result of the quantitative phytochemistry is shown in Table 2. We find all the classes of chemical compounds demonstrated in RSE but in different amounts. Indeed, RSE contains 36.35 ± 1.06 mg GAE/g of dry extract of total phenols, 11.91 ± 0.15 mg QE/g of dry extract of total flavonoids, and 13.01 ± 0.24 mg CE/g of dry extract of total tannins. There are more total phenols in the RSE than the total flavonoids and tannins.

### 3.3. Free Radical Scavenging Potential of RSE

The anti-free radical activity of RSE is shown in Tables 3 and 4. It appears that the RSE has a lower DPPH scavenging activity (IC_50_ = 17.51 ± 0.68 *μ*g/mL) than that of Trolox (IC_50_ = 22.48 ± 0.16 *μ*g/mL) (Table 3). In addition, the anti-free radical ABTS activity of the RSE (IC_50_ = 21.89 ± 0.39 *μ*g/mL) was also lower than that of BHA (IC_50_ = 23.05 ± 0.77 *μ*g/mL) (Table 4).

### 3.4. Inhibitory Effects of RSE on the *α*-Amylase Activity

**Figure 1:**
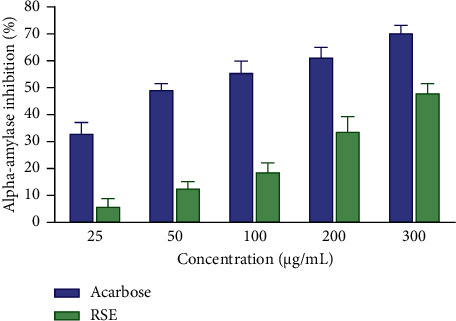
*α*-Amylase inhibitory activity of the RSE and acarbose. Each result represents the mean ± standard derivation (*n* = 3). Data analysis was performed by two-way ANOVA followed by Bonferroni's post hoc test. RSE: *Rytigynia senegalensis* extract.

Figure 1 shows the inhibitory effect of RSE on the *α*-amylase activity. Observations reveal a gradual change in the percentage inhibition depending on the concentration. In fact, acarbose showed significant inhibitory potential with inhibition percentages varying between 33.29% and 70.60%, corresponding to concentrations ranging from 25 to 300 *μ*g / mL, i.e., an average inhibitory concentration (IC_50_) of 69.47 *μ*g /mL (Table 5). RSE, for its part, showed inhibitory activity of 6.15% and 48.36% for respective concentrations of 25 and 300 *μ*g/mL, i.e., an IC_50_ of 308.83 *μ*g/mL (Table 6).

### 3.5. Inhibitory Effect of RSE on the *α*-Glucosidase Activity

**Figure 2:**
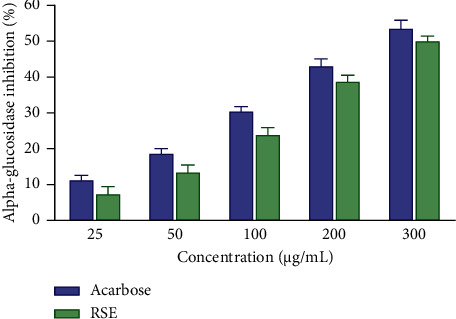
*α*-Glucosidase inhibitory activity of the RSE and acarbose. Each result represents the mean ± standard derivation (*n* = 3). Data analysis was performed by two-way ANOVA followed by Bonferroni's post hoc test. RSE: *Rytigynia senegalensis* extract.

Figure 2 shows the inhibitory effect of RSE on the activity of the *α*-glucosidase enzyme. Acarbose caused a strong inhibition of *α*-glucosidase activity with percentages of 11.48 and 53.77 for the respective concentrations of 25 and 300 *μ*g/mL, i.e., an IC_50_ of 284.23 *μ*g/mL (Table 5). Likewise, RSE also has significant inhibitory potential on *α*-glucosidase activity. This inhibition varies from 7.56% to 50.29% for concentrations ranging from 25 to 300 *μ*g/mL, i.e., an IC_50_ of 354.13 *μ*g/mL (Table 6).

### 3.6. Effects of RSE during the Starch Tolerance Test

**Figure 3:**
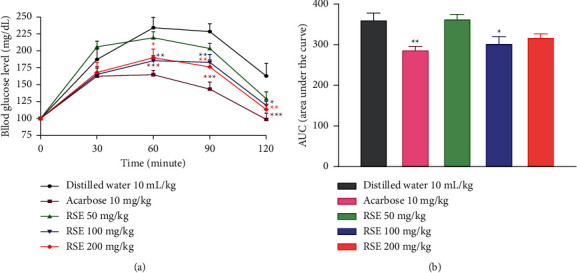
Effects of the RSE on the variation of glycemia (a) and AUC (b) during the starch tolerance test in normal rats. Each result represents the mean ± standard derivation (*n* = 6). Data analysis was performed by two-way ANOVA followed by Bonferroni's post hoc test. ^*∗*^*p* < 0.05; ^*∗∗*^*p* < 0.01; ^*∗∗∗*^*p* < 0.001 compared with the control group. RSE: *Rytigynia senegalensis* extract.

Figure 3 shows the results of the starch tolerance test in rats with temporary hyperglycemia treated with different doses of RSE. It can be seen from this figure that the blood glucose levels of all the animals increased at the 30th min and then gradually decreased until the end of the experiment. However, the blood glucose levels of the animals treated with acarbose and the various doses of RSE remained lower than that of the rats in the control group. Compared with animals in the control group, acarbose caused a significant drop (*p* < 0.001) in blood glucose levels at the 60th, 90th, and 120th minutes. Likewise, 200 mg/kg dose of RSE caused a significant reduction in blood glucose levels at the 60 (*p* < 0.05), 90 (*p* < 0.01), and 120th min (*p* < 0.01) posttreatment. A significant decrease in blood glucose level was also noted at the 60 (*p* < 0.01), 90 (*p* < 0.01), and 120th min (*p* < 0.05) with the dose of 100 mg/kg of RSE.

Compared with rats in the control group, acarbose resulted in a significant (*p* < 0.01) reduction in AUC. Furthermore, only the 100 mg/kg dose of RSE also caused a significant decrease (*p* < 0.05) in AUC (Figure 3).

### 3.7. Effects of RSE during the Sucrose Tolerance Test

**Figure 4:**
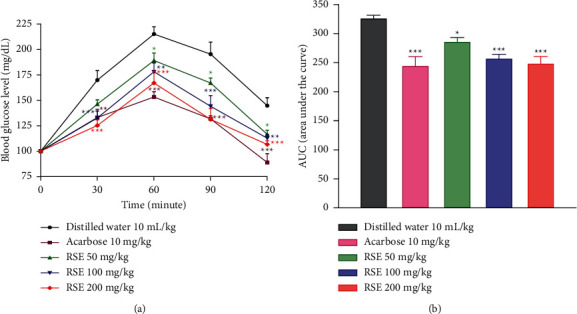
Effects of the RSE on the variation of glycemia (a) and AUC (b) during the sucrose tolerance test in normal rats. Each result represents the mean ± standard derivation (*n* = 6). Data analysis was performed by two-way ANOVA followed by Bonferroni's post hoc test. ^*∗*^*p* < 0.05; ^*∗∗*^*p* < 0.01; ^*∗∗∗*^*p* < 0.001 compared with the control group. RSE: *Rytigynia senegalensis* extract.

RSE and acarbose lowered postprandial blood glucose levels at all time slots, compared with the control group (Figure 4). In fact, there is a significant decrease (*p* < 0.01 to *p* < 0.001) in blood glucose levels at the 30, 60, 90, and 120th minutes after administration of acarbose and RSE at doses of 100 and 200 mg/kg, compared with the control group. Moreover, at the dose of 50 mg/kg of RSE, the decrease (*p* < 0.05) in blood glucose was observed rather at the 60th, 90th, and 120th minutes.

Both acarbose and RSE at doses of 100 and 200 mg/kg induced a significant (*p* < 0.001) decrease in AUC, compared with the control group (Figure 4). This decrease was less significant (*p* < 0.05) with the dose of 50 mg/kg of RSE.

### 3.8. Hypoglycemic Plus Antihyperglycemic Effects of RSE

The effects of the different treatments on baseline glycemia plus oral-induced hyperglycemia in normal rats are shown in Table 7. It appears from this table that no significant change in glycemia was noted at the 30th, 60th, and 90th minutes after administration of RSE at all doses, compared with the control group. On the other hand, a significant fall (*p* < 0.001) in the blood glucose level was noted in the rats having received glibenclamide at the 60th and 90th minutes.

In addition, 90 minutes after the carbohydrate overload, blood sugar levels fell significantly (*p* < 0.01) with glibenclamide from the 120th to the 180th minutes, compared with the control group. Similarly, at the 150th and 180th minutes, the administration of RSE at doses of 100 and 200 mg/kg resulted in a significant (*p* < 0.01) drop in blood sugar level.

## 4. Discussion

Inhibiting the activity of *α*-amylase and *α*-glucosidase, enzymes involved in the digestion of carbohydrates, can significantly reduce the rise in postprandial blood glucose and therefore may be an important strategy in the management of blood sugar levels in type 2 diabetics [30]. Unfortunately, current treatments are limited only to poor control of the exacerbation, and in addition, there are various harmful side effects. However, natural inhibitors of *α*-amylase and *α*-glucosidase activity contained in various plants have fewer or no side effects [31]. *α*-Amylase enzymes hydrolyze complex polysaccharides to produce oligosaccharides and disaccharides, which are then hydrolyzed by *α*-glucosidase enzymes to monosaccharides, which are absorbed by the intestines [32]. *α*-Amylase and *α*-glucosidase enzyme inhibitors delay digestion and absorption of carbohydrates, thereby lowering postprandial glucose levels [33].

Medicinal plants and their fruits/seeds including cinnamon, aloe, gymnema, bitter melon, coffee, guava, cocoa, green tea, nettle, garlic, sage, caper, soybean, turmeric, yerba mate, and walnut have got tremendous focus and have scientifically proved antidiabetic potentials. Several isolated compounds from natural origin including phenolic, galegine, pycnogenol, miglitol, voglibose, and acarbose are extensively employed for the management of T2DM [34].

*In vitro* studies have shown the inhibitory effect of acarbose and RSE on the activity of *α*-amylase and *α*-glucosidase enzymes. Based on the IC_50_ values, the inhibitory potency of the RSE was stronger on the *α*-amylase enzyme than on the *α*-glucosidase. Concentration-dependent inhibition was also observed with acarbose on both *α*-glucosidase enzyme activity and *α*-amylase enzyme activity. These results are similar to those of Mahamad et al. [35] who showed that the aqueous extract of *Cissus polyantha* has a strong inhibitory effect on the activity of *α*-amylase and *α*-glucosidase.

However, the inhibitory activity *in vitro* is not always linked to the corresponding *in vivo* activity. Thus, the results obtained *in vitro* must be proven *in vivo* studies. It is with this in mind that in vivo studies on starch and sucrose tolerance tests were carried out. Thus, in these studies, doses of 100 and 200 mg/kg of RSE produced a significant decrease in blood glucose levels at the 60, 90, and 120th minutes after administration of the starch. Likewise, 100 mg/kg dose significantly lowered AUC. For the sucrose tolerance test, all doses of the RSE significantly lowered AUC and blood glucose levels at the 60, 90, and 120th minutes. RSE appears to delay the rapid digestion of starch and sucrose to increase the duration of carbohydrate absorption and thereby lower postprandial hyperglycemia. Thus, RSE showed a significant inhibitory effect on both types of *α*-amylase and *α*-glucosidase enzymes *in vitro* and *in vivo*. These results suggested that the RSE could decrease postprandial glucose levels by inhibiting the activity of *α*-amylase and *α*-glucosidase, which are enzymes important in the digestion of carbohydrates [36]. Polyphenols and flavonoids from plants are known as natural antidiabetic agents having an inhibitory effect on the activity of digestive enzymes responsible for the hydrolysis of carbohydrates [37, 38]. Our results show the presence of total phenols (36.35 mg GAE/g of extract), flavonoids (11.91 mg QE/g of extract), and tannins (13.01 mg CE/g of extract). The presence of these compounds in the aqueous extract would be responsible for the antihyperglycemic effects *in vitro* and *in vivo* observed in the present work.

In this study, the ability of the RSE to donate hydrogen was verified using the radicals DPPH and ABTS. The amount of sample required to decrease the initial concentration of free radical by 50% is a parameter widely used to measure the anti-free radical activity of plant extracts. Indeed, the anti-free radical potential of the plant is inversely proportional to the average inhibitory concentration (IC_50_). The results of our study showed significant radical scavenging activity on DPPH (IC_50_ = 21.89 *μ*g/mL) and ABTS (IC_50_ = 14.65 *μ*g/mL) radicals. Phenolic compounds have the ability to scavenge free radicals thanks to their hydroxyl groups [39]. They mainly act as reducing agents, hydrogen donors, singlet oxygen extinguishers, or metal chelators [40]. These different classes of chemical compounds present in RSE would be responsible for the anti-free radical potential observed in this study.

During the glucose tolerance test, glibenclamide and RSE (100 and 200 mg/kg) led to a significant reduction in postprandial hyperglycemia from the 120th minute, compared with the control group. Indeed, glibenclamide is a sulfonylurea antidiabetic that stimulates the secretion of insulin by binding to the protein (sulfonylurea) of the channels (K + -ATP) of the beta cells of the islets of Langerhans causing their depolarization with, as a consequence, an entry of the calcium in beta cells, responsible for the exocytosis of insulin storage granules [41]. RSE would have acted either by stimulating the secretion of insulin-like reference substance or by mimicking the effects of insulin at the level of its specific receptors or by decreasing gluconeogenesis and glycogenolysis or by increasing the use of glucose and glycolysis [42, 43]. The antihyperglycemic activity observed in this work would be due to a synergistic action of the bioactive compounds (phenols, flavonoids, and tannins) present in RSE.

## 5. Conclusion

RSE has anti-free radical and inhibitory properties on the activity of *α*-amylase and *α*-glucosidase enzymes and therefore reduces postprandial glycemia. These pharmacological effects would be due to the presence of bioactive compounds in the RSE. In future work, we will study different models of diabetes and its comorbidities to accurately confirm the antidiabetic properties of RSE.

## Figures and Tables

**Table 1:** Qualitative phytochemical screening.

Chemical compounds	RSE
Tannins	+
Phenols	+
Flavonoids	+
Saponins	+
Quinones	+
Terpenoids	+
Steroids	−
Alkaloids	−
Glycosides	+

+: presence, −: absence.

**Table 2:** Total phenol, flavonoid, and tannin contents of RSE.

Chemical compounds	Total phenols (mg gallic acid E/g extract)	Total flavonoids (mg quercetin E/g extract)	Total tannins (mg catechin E/g extract)
RSE	36.35 ± 1.06	11.91 ± 0.15	13.01 ± 0.24

Each result represents the mean ± standard derivation (*n* = 3). Data analysis was performed by one-way ANOVA followed by Tukey's post hoc test. E: equivalent; RSE: *Rytigynia senegalensis* extract.

**Table 3:** Anti-free radical DPPH activity of RSE.

Group	Concentration (*μ*g/mL)	Inhibition (%)	IC_50_ (*μ*g/mL)
RSE	10	1.71 ± 0.02	17.51 ± 0.68
	20	42.74 ± 0.37	
	30	49.98 ± 0.65	
	40	52.31 ± 0.72	
	50	56.65 ± 0.44	
	60	69.69 ± 0.57	

Trolox	10	1.72 ± 0.03	22.48 ± 0.16
	20	30.13 ± 0.25	
	30	60.20 ± 0.50	
	40	76.57 ± 0.68	
	50	73.60 ± 0.71	
	60	75.05 ± 0.48	

Each result represents the mean ± standard derivation (*n* = 3). Data analysis was performed by one-way ANOVA followed by Tukey's post hoc test. IC_50_: inhibitor concentration to inhibit 50% of the activity under the assayed conditions. DPPH: 2,2-diphenyl-1-picrylhydrazyl. RSE: *Rytigynia senegalensis* extract.

**Table 4:** Anti-free radical ABTS activity of RSE.

Group	Concentration (*μ*g/mL)	Inhibition (%)	IC_50_ (*μ*g/mL)
RSE	10	1.71 ± 0.02	21.89 ± 0.39
	20	30.74 ± 0.26	
	30	52.98 ± 0.41	
	40	55.31 ± 0.55	
	50	59.65 ± 0.38	
	60	64.89 ± 0.25	

Butylhydroxyanisole	10	1.72 ± 0.05	23.05 ± 0.77
	20	24.13 ± 0.33	
	30	52.98 ± 0.60	
	40	64.56 ± 0.77	
	50	72.60 ± 0.49	
	60	75.05 ± 0.64	

Each result represents the mean ± standard derivation (*n* = 3). Data analysis was performed by one-way ANOVA followed by Tukey's post hoc test. BHA: butylhydroxyanisole. IC_50_: inhibitor concentration to inhibit 50% of the activity under the assayed conditions. ABTS: 2,2-azino-bis-3-ethyl benzothiazoline-6-sulphonic acid. RSE: *Rytigynia senegalensis* extract.

**Table 5:** Percentage inhibition of RSE on the *α*-amylase activity and IC_50_ values.

Group	Concentration (*μ*g/mL)	Inhibition (%)	IC_50_ (*μ*g/mL)
RSE	25	6.15 ± 5.41	308.93 ± 13.06
	50	12.94 ± 4.50	
	100	18.96 ± 6.20	
	200	34.04 ± 10.47	
	300	48.36 ± 6.44	

Acarbose	25	33.29 ± 7,62	69.47 ± 5.27
	50	49.49 ± 3.98	
	100	55.90 ± 8.01	
	200	61.55 ± 6.79	
	300	70.60 ± 5.14	

Each result represents the mean ± standard derivation (*n* = 3). Data analysis was performed by one-way ANOVA followed by Tukey's post hoc test. IC_50_: concentration of the extract necessary to inhibit the activity of the enzyme by 50%. RSE: *Rytigynia senegalensis* extract.

**Table 6:** Percentage inhibition of RSE on the *α*-glucosidase activity and IC_50_ values.

Group	Concentration (*μ*g/mL)	Inhibition (%)	IC_50_ (*μ*g/mL)
RSE	25	7.56 ± 3.76	354.13 ± 14,71
	50	13.66 ± 3.63	
	100	24.12 ± 3.62	
	200	38.95 ± 3.18	
	300	50.29 ± 2.25	

Acarbose	25	11.48 ± 2.19	284.23 ± 11,59
	50	18.89 ± 2.25	
	100	30.66 ± 2.19	
	200	43.31 ± 3.63	
	300	53.77 ± 4.15	

Each result represents the mean ± standard derivation (*n* = 3). Data analysis was performed by one-way ANOVA followed by Tukey's post hoc test. IC_50_: concentration of the extract necessary to inhibit the activity of the enzyme by 50%. RSE: *Rytigynia senegalensis* extract.

**Table 7:** Hypoglycemic plus antihyperglycemic effect of RSE.

Groups	Blood glucose levels (mg/dL)						
	0 min	30 min	60 min	90 min (3 g/kg D-glucose)	120 min	150 min	180 min
Control	49.5 ± 4.1	52.5 ± 2,2	40.0 ± 1.4	66.2 ± 1.3	68.5 ± 1.6	55.7 ± 2.7	49.0 ± 3.0
Glibenclamide	51.2 ± 2.1	51.2 ± 1.8	25.5 ± 2.2^*∗∗∗*^	45.7 ± 4.5^*∗∗∗*^	33.7 ± 2.6^*∗∗∗*^	25.5 ± 1.2^*∗∗∗*^	23.0 ± 1.1^*∗∗∗*^
RSE 50 mg/kg	44.0 ± 2.7	52.5 ± 2.9	34.0 ± 1.2	60.0 ± 3.8	59.2 ± 1.2	45.0 ± 5.8	40.2 ± 2.9
RSE 100 mg/kg	47.5 ± 2.2	53.0 ± 2.7	32.0 ± 3.2	65.7 ± 6.6	59.2 ± 5.5	39.7 ± 1.8^*∗∗*^	32.7 ± 2.4^*∗∗*^
RSE 200 mg/kg	51.2 ± 2.1	61.5 ± 1.5	40.7 ± 3.2	72.5 ± 3.1	64.2 ± 5.8	41.7 ± 3.2^*∗*^	31.5 ± 0.3^*∗∗*^

Each result represents the mean ± standard derivation (*n* = 6). Data analysis was performed by two-way ANOVA followed by Bonferroni's post hoc test. ^*∗*^*p* < 0.05; ^*∗∗*^*p* < 0.01; ^*∗∗∗*^*p* < 0.001 compared with the control group. RSE: *Rytigynia senegalensis* extract.

## Data Availability

The experimental data used to support the findings of this study can be obtained from the corresponding author upon request.

## Supplementary Materials

Supplementary Materials Supplementary Materials. Standard curves of gallic acid E/g extract, quercetin E/g extract, and catechin E/g extract. Figure 1. Standard curve of gallic acid. Figure 2. Standard curve of quercetin. Figure 3. Standard curve of catechin.
