# Supplementary material for: Efficacy of Rytigynia senegalensis Blume on Free Radical Scavenging, Inhibition of α-Amylase and α-Glucosidase Activity, and Blood Glucose Level
Source: Evid Based Complement Alternat Med. 2022 Sep 27;2022:9519743. doi: 10.1155/2022/9519743 (PMC9532085; doi:10.1155/2022/9519743)
Supplement: Supplementary Materials — Standard curves of gallic acid E/g extract, quercetin E/g extract, and catechin E/g extract. Figure 1. Standard curve of gallic acid. Figure 2. Standard curve of quercetin. Figure 3. Standard curve of catechin. [file 9519743.f1.docx]

**Standard curves of Gallic acid E/g extract and Quercetin E/g extract, Catechin E/g extract**

Figure 1 : Standard curve of Gallic acid

Figure 2 : Standard curve of quercetin

Figure 3 : Standard curve of catechin
